# Application of stem cells combined with biomaterial in the treatment of intervertebral disc degeneration

**DOI:** 10.3389/fbioe.2022.1077028

**Published:** 2022-11-25

**Authors:** Zongtai Liu, Yuya Bian, Guangzhi Wu, Changfeng Fu

**Affiliations:** ^1^ Department of Spine Surgery, First Hospital of Jilin University, Changchun, China; ^2^ Department of Orthopedics, Affiliated Hospital of Beihua University, Jilin, China; ^3^ Jilin Institute of Scientific and Technical Information, Changchun, China; ^4^ Department of Hand Surgery, China-Japan Union Hospital of Jilin University, Changchun, China

**Keywords:** intervertebral disc, intervertebral disc degeneration, low back pain, stem cell, biomaterial, regenerative medicine

## Abstract

As the world population is aging, intervertebral disc degeneration (IDD) is becoming a global health issue of increasing concern. A variety of disc degeneration diseases (DDDs) have been proven to be associated with IDD, and these illnesses have significant adverse effects on both individuals and society. The application of stem cells in regenerative medicine, such as blood and circulation, has been demonstrated by numerous studies. Similarly, stem cells have made exciting progress in the treatment of IDD. However, due to complex anatomical structures and functional requirements, traditional stem cell injection makes it difficult to meet people’s expectations. With the continuous development of tissue engineering and biomaterials, stem cell combined with biomaterials has far more prospects than before. This review aims to objectively and comprehensively summarize the development of stem cells combined with contemporary biomaterials and the difficulties that need to be overcome.

## 1 Introduction

The intervertebral disc (IVD) is the cartilaginous tissue that lies between the spinal segments and plays a crucial role in maintaining the normal physiological activity of the spine. With the increase in aging populations worldwide, intervertebral disc degeneration (IDD) is becoming a health problem that is difficult to ignore or compromise. IDD could lead to disc degeneration diseases (DDDs), such as compression of blood vessels or nerves, and disc herniation (Kim H. S. et al., 2020). DDDs often cause lower back pain and may be associated with motor dysfunction and sensory abnormalities (Luoma et al., 2000). In the United States alone, back problems account for more than seven million emergency department visits and more than two million hospital admissions per year (Owens et al., 2006). In social and economic terms, the direct cost of health care and the indirect economic losses due to pain is still staggering. In 2016, lower back and neck pain accounted for the highest medical expenditure of around $ 134.5 billion among 154 medical conditions included in the United States (Dieleman et al., 2020).

The specific causes of disc degeneration are not fully understood. The prevailing view is that IDD is a combination of factors, such as age, genetics, and diabetes (Rider et al., 2019; Francisco et al., 2022). Typical pathological features of IDD include decreased water content and height of the disc, disturbed balance between synthesis and decomposition of the extracellular matrix (ECM), continuous loss of cells, and changes in the microenvironment of the IVD tissue (Binch et al., 2021). At present, the clinical treatment of DDD can be roughly categorized as either conservative or surgical. Common conservative treatments include analgesics, appropriate functional exercise, acupuncture, and massage. Conservative management may be effective in patients with mild symptoms or early-stage IDD (Corp et al., 2021). Surgery is still the only option for patients who do not respond to conservative treatment or who might have developed severe complications. The most frequently used procedures include discectomy and fusion. In recent years, the rapid development of endoscopic and postoperative rapid rehabilitation technologies has greatly shortened the length of hospital stays, in addition to reducing the possibility of many postoperative complications. However, the biomechanical changes that are brought by surgery to the overall spine are still irreversible (Ahn, 2019; Gadjradj et al., 2021). The abnormal mechanical structure will lead to further aggravation of the load on the adjacent segments, which further expands the possibility of disc degeneration of the adjacent spinal segments. In addition, the persistent neurological symptoms during long-term follow-up and the possibility of reoperation prove that surgery is not a one-and-done option (Lurie and Tomkins-Lane, 2016; Wu et al., 2021; Katz et al., 2022). In conclusion, all current treatments focus on the remedy and resolution of existing symptoms. Earlier interventions on the IDD pathological process or on the reversal of the degeneration process have received extensive attention and a lot of exploration research has been carried out in recent years.

Many promising therapeutic approaches have been developed for the various mechanisms of the IDD, such as gene therapy, biological agents, and growth factors. Some of them have been in pre-clinical trials while others have been approved for clinical use (Kamali et al., 2021). However, the treatment for single pathogeny is often difficult to meet people’s expectations in the treatment of IDD cases that are caused by multiple pathological processes. Due to the easier availability, stronger differentiation ability and extremely low immunogenicity of stem cells, these cells can target multiple pathological pathways (Zakrzewski et al., 2019; Bhujel et al., 2022). The successful application of stem cells in blood and circulatory related diseases seems to further raise the hopes for the possible application of stem cell therapy for IDD. However, even without external factors such as ethical approval, the complex anatomical structure and functional requirements make stem cells difficult to retain, not to talk about surviving in the proposed location, which undoubtedly limits further exploration of stem cell therapy for IDD. With the continuous development of tissue engineering and biomaterials, stem cells combined with biomaterials have shown promising application prospects. Biomaterials do not only provide a suitable physical and chemical environment for stem cells, but also carry drugs or cytokines, to promote better directional differentiation of the stem cells. The designed biomaterials can also have a good immobilization effect on the stem cells, in addition to preventing the leakage of the cells. This review aims to provide an overview of the current advances in the treatment of IDD with stem cells combined with biomaterials. We will also discuss the selection of stem cells and biomaterials. Finally, we will summarize the current limitations and future challenges that are associated with treatment of IDD.

## 2 Anatomy of intervertebral disc

**FIGURE 1:**
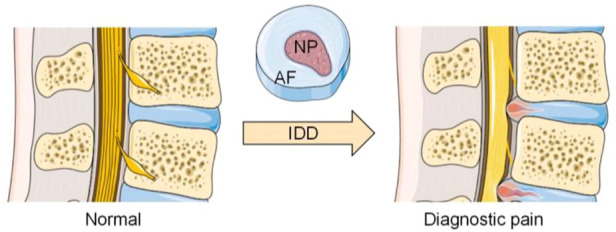
Anatomical changes between normal and degenerated discs. (The Figure was partly generated using Servier Medical Art, provided by Servier, licensed under a Creative Commons Attribution 3.0 unported license).

IVD is the largest avascular tissue in the human body, which is mainly composed of nucleus pulposus (NP), annulus fibrosus (AF) and cartilage endplate (CEP) (Roberts et al., 2006; Smith et al., 2011). Due to its avascular nature, it has a poor ability to repair itself after injury and degeneration. To better understand the mechanism of stem cell therapy for IDD, it is necessary to understand the main physiological characteristics and functional requirements of each part in IVD (Figure 1).

### 2.1 The nucleus pulposus

The NP is the colloidal part that is located at the center of the IVD. The NP contains more than 70% water, which reduces the effects of external pressure and prevents internal stress concentration (Raj, 2008). The remaining components of the NP are mainly proteoglycan and collagen. Aggrecan is the most abundant type of proteoglycan in NP, though other forms such as biglycan and versican are also present (Melrose et al., 2001). Proteoglycans are essential for maintaining the high-water content of NP. There is type Ⅰ and type II collagen. The NP has a higher content of type II collagen than other IVD tissues (Raj, 2008). The NP cells are present in a colloid-like matrix that is composed of proteoglycan and collagen. In general, the cell density in the NP tissue is relatively low, only 2–5*10^6^ cells/ml, and it further decreases with age (Maroudas et al., 1975; Stairmand et al., 1991). In the early stage of IVD development, the NP has vascular distribution and notochord-derived cells (Roberts et al., 2006; de Bree et al., 2018), both of which are not found in adult NPs (Colombini et al., 2008). The presence of notochord-derived cells has been linked to self-repair abilities, while the lack of vascular distribution means that NP cells are in a relatively anoxic environment, so nutrition or drugs are difficult to transport through the blood (Risbud and Shapiro, 2011; Boubriak et al., 2013). All these suggest that NP has a fragile ability to repair itself.

As mentioned above, proteoglycans and high osmotic pressure are essential for resisting axial compressive forces and spinal pressure during daily motion and activities. The range of mechanical loads required by the NP is enormous. When lying prone, the pressure load on the NP is only 0.1 MPa, but when bending down to lift heavy objects, the pressure load will rise to 2.3 MPa (Schultz et al., 1982; Adams et al., 1996). Abnormal mechanical loads have been proved to be an important factor that contributes to IDD (Vergroesen et al., 2015). The main pathological changes in the NP are proteoglycan loss, type II collagen and osmolality reduction. In spine magnetic resonance imaging (MRI), these changes manifest as decreased disc and appear darkened to black on the T2 signal (Antoniou et al., 1996; Brinjikji et al., 2015).

### 2.2 The anulus fibrosus

The AF is the fibrocartilage that surrounds the NP, and can be further divided into outer and inner AF (Hsieh and Twomey, 2010). The main function of the AF is to stabilize and encapsulate the NP. In the presence of an intact AF structure, the NP tissue can maintain a high hydrostatic pressure, and the entire disc can resist the high intensity pressure that emanates from daily motion such as flexion-extension and lateral bending. The AF is a lamellar structure that is predominantly composed of type Ⅰ collagen in a highly oriented manner, with about 15–25 layers (Marchand and Ahmed, 1990). As the AF approaches the NP, the proportion of type II collagen and water content gradually increases (Feng et al., 2006). The cell density in the AF is higher than that in the NP, with a range of about 5–10*10^6^ cells/ml (Bowles and Setton, 2017). These cells are arranged in concentric lamellae that interleave each other and are offset by 30–60° from the vertical spinal axis (Walker and Anderson, 2004; Raj, 2008). During the early stages of IVD development, the boundary between the NP and AF is not obvious but as the development approaches maturity, clear morphological boundary begins to appear (Roberts et al., 2006).

When the AF structure is damaged or fractured, the uneven distribution of stress may lead to the herniation or displacement of the IVD tissue, which may further compress the surrounding blood vessels and nerves, thereby causing obvious clinical symptoms. Despite the possibility of adequate decompression by surgical treatment, the AF structure is not repaired, and the abnormal biomechanical structure is not effectively corrected. Although AF and NP are significantly different in composition and structure, they are closely related in maintaining normal physiological functions. Moreover, any tiny damage on any single part will lead to the disorder of the overall function.

### 2.3 The cartilage endplate

The CEP is composed of the hyaline cartilage and lies between the soft tissue of the IVD and the bony structure of the vertebral body (Luo et al., 2021). The hyaline cartilage is mainly composed of water, proteoglycans, and collagen. The CEPs are critical for maintaining the mechanical integrity, as well as the vascular and avascular separation of the IVDs. Due to the avascular structure of the IVD, the essential substances such as glucose and oxygen are mainly supplied by the capillaries that are around the IVD through CEP, to maintain cell activity (Urban et al., 2004; Malandrino et al., 2014; Bowles and Setton, 2017). The CEP in adults is thinner and less vascularized than the endplates in the early development stage (Harmon et al., 2020). Comparable to osteoarthritis, cartilage damage is also one of the pathological features of IDD (Francisco et al., 2022). Thinning and mineralized CEP not only makes it difficult to maintain spinal biomechanical stability, but also prevent nutrients from entering the NP and AF through the CEP (Huang et al., 2014). All these factors will further aggravate the rate of intervertebral disc degeneration. In addition to these, the increase in nerve fibers in the CEP of the degenerative segment is also thought to be related to the pain that is caused by IDD. In recent years, promoting CEP repair in a bid to enhance the nutrient supply to the IVD has become an alternative way to interfere with the progression of IDD. Having said this, stem cells have also achieved promising application prospects in promoting cartilage regeneration (Yang et al., 2018; Kangari et al., 2020).

## 3 Applications of stem cells in combination with biomaterials

### 3.1 Types and selection of stem cells and biomaterials

#### 3.1.1 Selection of stem cells

Overall, stem cells have the advantages of low immunogenicity, strong ability to induce differentiation, and tolerance to hypoxia and low glucose. There are also some differences in the function, survival, and acquisition of different types of stem cells. Choosing the right type of stem cell is undoubtedly an important step in using stem cells to treat IDD (Table.1). At present, stem cells can be divided into mesenchymal stem cells (MSCs), pluripotent stem cells (PSCs), and IVD-derived stem cells (IVDSCs). According to their source, MSCs can be further divided into umbilical cord mesenchymal stem cells (UCMSCs), adipose-derived mesenchymal stem cells (ADMSCs), and bone marrow mesenchymal stem cells (BMSCs) (Zhang et al., 2022). UCMSCs are relatively young stem cells with excellent differentiation potential, and because they are self-provided and the means of acquisition are non-invasive, there is no need to consider ethical barriers (Chen et al., 2013; Garzón et al., 2014). Unfortunately, the retention and acquisition time of UCMSCs is short. Additionally, IDD patients lose the opportunity to obtain autologous UCMSCs when symptoms are obvious. Another thing to note is that the experimental cost of UCMSCs is high yet the treatment outcome is not significantly different from the results of using other stem cells. Considering the wide distribution and easy availability of adipose tissue in the human body, ADMSCs seem to be an ideal choice. A large number of *in vivo or in vitro* experiments have proved that ADMSCs can intervene or even reverse the pathological process of IDD (Lu et al., 2008; Jin et al., 2013; Clarke et al., 2014). Unfortunately, the endochondral osteogenesis ability of ASMCS is not satisfactory (Diekman et al., 2010). At present, BMSCs have attracted the most attention in stem cell therapy for IDD. BMSCs are non-hematopoietic stem cells that are located in the bone marrow. These stem cells are characterized by an ideal differentiation potential and self-renewal attributes. Although many previous studies that used animal models and some small clinical cohort studies have pointed out that BMSCs have exciting application prospects in the treatment of IDD (Cao et al., 2015; Kumar et al., 2017; Teixeira et al., 2018), the method for obtaining these stem cells is invasive. Also, there is a lack of long-term clinical cohort observation so the application of BMSCs in the treatment of IDD still needs to be carefully evaluated. PSCs include embryonic stem cells (ESCs) and induced pluripotent stem cells (IPSCs). Considering that ESCs are extracted from frozen embryos, their clinical application is subject to strict ethical restrictions. IPSCs can differentiate into NP cells in the ECM after receiving some inducing factors that are secreted by NP cells (Liu et al., 2015b). It is worth noting that the proliferation and differentiation patterns of IPSCs are similar to tumor cells, which makes them potentially carcinogenic. In recent years, the method of avoiding carcinogenicity using IPSC-based exosomes has gradually received attention (Qi et al., 2016). Comparable to MSCs, IVDSCs can be classified according to their origin from NP, AF, and CEP. Although these native tissue-derived stem cells seem to have a broad application prospect, the low separation efficiency and harsh microenvironment in the degenerated IVD hinder the further exploration of IVDSCs.

**TABLE 1:** Advantages and disadvantages of various stem cells.

Cell subtype	Cell source	Major advantage	Major disadvantages
MSC			
UCMSC	umbilical cord	Autologous cells have low immunogenicity and can avoid ethical problems; near-ideal potential for proliferation and differentiation	It is very difficult to obtain human UCMSC due to the special existence period
ADMSC	adipose tissue	A wide range of cell sources; cell can be obtained noninvasively; autologous cells have low immunogenicity and can avoid ethical problems	Cartilage and bone regeneration capacity is not as good as BMSC
BMSC	bone marrow	Autologous cells have low immunogenicity and can avoid ethical problems; mature preparation technology; strong cartilage regeneration capacity	The acquisition method is invasive operation
PSC			
ESC	early embryos	Near-ideal potential for proliferation and differentiation	Cell sources are scarce and face ethical issues
IPSC	Reprogramming differentiated somatic cells	Strong proliferation and differentiation ability, cells can be derived from patients, low immunogenicity	May integrate viral or oncogenic external genes
IVDSC			
NPSC	NP	Present in NP tissue inherently; promotes self-renewal of the NP tissue and no external cells are needed	The cell stock is low; difficulty in isolating cells; limited capacity for proliferation and differentiation
AFSC	AF	Present in AF tissue inherently; sensitive to the mechanical environment	The cell stock is low; difficulty in isolating cells; limited capacity for proliferation and differentiation
CEPSC	CEP	Strong osteogenesis and chondrogenesis ability; promote the proliferation of other cells	The cell stock is low; difficulty in isolating cells; limited capacity for proliferation and differentiation

#### 3.1.2 Selection of biomaterials

Compared with the limited selection of stem cell types, the options for materials have become more diversified due to the continuous development of materials through science and industrial technology. However, multiple options do not always simplify the problem; they sometimes even make it more complex. Considering the load-bearing requirements of a normal disc, the materials need to simulate the mechanical properties of normal tissue as much as possible (Manoukian et al., 2018; Zhu et al., 2021). In addition, targeted selection of biocompatibility such as cell adhesion should be made according to the characteristics of the corresponding tissue. Finally, cytotoxicity, immunogenicity, degradability, and manufacturing cost are inescapable problems for all implants. At present, the choice of materials is often a trade-off between the above-mentioned properties, to a certain extent. When one property is improved, another performance is usually compromised (Table 2). For example, in the selection of scaffold materials, it is often difficult to have both remarkable mechanical properties and porosity.

**TABLE 2:** A brief comparison of different biomaterials used to deliver stem cells.

Delivery vehicles	Major advantages	Major disadvantages
Hydrogel		
natural composition	Provide a suitable environment for cell survival; low immunogenicity and cytotoxicity	Mechanical properties are usually unsatisfactory
synthetic composition	Stimulus-response hydrogels have a good degree of tissue fit; Some parameters that can be adjusted manually	Potential cytotoxicity
Tissue derived scaffold	Similar to the composition of the intervertebral disc	Difficult to manufacture on a large scale; some parameters are difficult to adjust manually
Microsphere	Injectability results in a good tissue fit; controlled release of the carried drug allows better control of the microenvironment	difficult to provide mechanical support
Artificial polymer materials		
Synthetic scaffold mimicking the IVD structure	Provides spatial structure and mechanical support similar to health IVD	Lack of ability to interfere with the microenvironment
Synthetic scaffolds loaded with therapeutic substances	Provides mechanical support and regulates the microenvironment at the same time	Difficult to design and the high manufacturing cost

Hydrogels are widely used to deliver cells due to their excellent water content, which can provide a suitable microenvironment for cell survival. Hydrogels have various components that can be classified into natural and synthetic components. The natural ingredients include collagen, hyaluronic acid, chitosan, and alginate. Generally, natural components have good biocompatibility and low cytotoxicity, but their mechanical properties are often unsatisfactory. Some of the common synthetic components are poly (N-isopropyl acrylamide), poly (ethylene glycol), and poly (lactic-co-glycolic acid) copolymers. These materials can be industrially produced in large quantities and have strong mechanical properties, but their cytotoxicity and immunogenicity should be considered. Composite hydrogels can combine components with different properties to achieve more ideal performance. However, the involvement of different components bring new problems to the overall material design. The *in vivo* safety of various combinations of components needs to be rigorously evaluated.

Several decellularized scaffolds have been developed. To better mimic the component composition of normal disc tissue. However, the scarcity of healthy donor sources that are suitable for humans limits their further development, and the use of animal-derived substitutes requires strict safety assessment. The microsphere system can control the release of substances in the delivery system in a more precise way, in addition to providing attachment sites for cells. Some of the common microsphere materials include natural materials such as gelatin, collagen and chitosan, as well as polylactic acid-glycolic acid, poly (L-lactic acid) and polycaprolactone. Unfortunately, it is difficult for the microspheres to provide mechanical support so other materials should be introduced, though this makes system design more difficult.

In recent years, some artificial polymer materials significantly improved the performance of biomaterials, with a remarkable balance in mechanical strength and porosity. Increased understanding of the structure of IVD and continuous improvement of the material preparation technology has made it possible to imitate the structure of tissues to provide a more suitable environment for stem cells. Additionally, scaffolds that are loaded with drugs or cytokines are better applicable cell differentiation and expression of corresponding products.

### 3.2 Replenish cells and improve the microenvironment

Most tissues and cells in the human body are undergoing constant renewal, and sufficient cells plays a crucial role in maintaining normal tissue function and metabolism. This rule also holds true for the IVD tissue. Again, it is worth noting that the low cell density, lack of a powerful source of regenerative cells, and the harsh microenvironment after IDD make the endogenous self-cell recruitment of IVD difficult. In degenerated IVD, cell loss was reflected by higher rates of senescence, apoptosis, and pyroptosis. Direct replacement of the missing cells seems to be the most straightforward and effective method, though there are still many limitations, such as the survival rate of the implanted cells and unnecessary cell dissipation. Stem cells combined with biomaterials offer new hope for stem cell-based endogenous repair.

Many experiments have shown that various stem cells can differentiate into functional cells of IVD if they are exposed to appropriate stimulation. Although conditions such as hypoxia are often considered to be unfavorable for cell survival, appropriate hypoxia is indeed a stimulating condition for further cell differentiation. Han *et al.* (Han et al., 2015), co-cultured human ligamentum derived stem cells and NP cells *in vitro* under hypoxia conditions and successfully differentiated ligamentum derived stem cells into NP-like cells. Strassburg *et al.* (Strassburg et al., 2010), reported varying stimulating effects of different NP cells. BMSCs were co-cultured with NP cells from degenerated or non -degenerated discs for 7 days. Co-culturing with NP cells derived from degenerated discs enhanced the expression of the transforming growth factor (TGF) -β and cartilage-derived morphogenetic protein -1, which can regulate NP cell metabolism and promote the production of ECM (Thompson et al., 1991). In an *in vivo* study using rabbit model, BMSCs were transplanted into degenerated IVD and resulted in significant improvement in cell numbers, cell survival rate, and disc water content after 24 weeks (Sakai et al., 2006). Similar findings have been reported in other types of stem cells. Zhao *et al.* (Zhao et al., 2020), isolated NP cells from patients with severe IDD and induced apoptosis by *in vitro* compression. After co-culturing with UCMSCs, the apoptosis was significantly reversed. Lu *et al.* (Lu et al., 2008), co-cultured ADMSCs with NP cells in a hydrogel containing collagen Ⅱ. The results showed that the number of cells that were producing type II collagen was significantly increased and the aggrecan-related genes were upregulated. In another *in vivo* study, when ESCs were induced for differentiation by TGF-β and ascorbic acid, they were implanted into the IDD model by needle puncture (Sheikh et al., 2009). The results revealed the presence of notochord-derived cells and higher proteoglycan content was observed. In recent years, notochord-derived cells have been identified as key points in IVD regeneration (Harfe, 2022). He *et al.* (He et al., 2018), also reported that co-culturing cartilage endplate stem cells (CESCs) with NP cells promoted the proliferation of NP cells.

**FIGURE 2:**
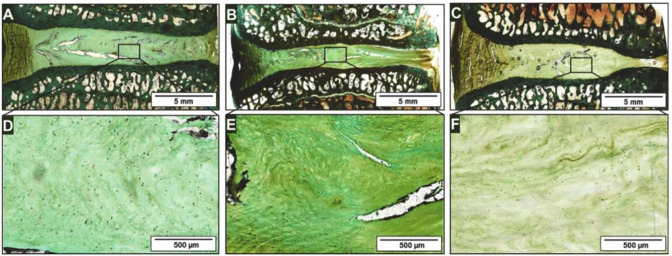
After 12 months of experiment, Masson-Goldner histological light microscopy was used to observe the representative IVD segments in a sheep model. The healthy segment view **(A)** and enlarged view of the NP region **(D)**; The IDD segment **(B)** and enlarged view of the NP region **(E)**; The IDD segment receiving scaffolds injection **(C)** and enlarged view of the NP region **(F)**. Reproduced with permission from (Friedmann et al., 2021).

Although the growth and differentiation of stem cells are necessary for the stem cell-based therapy, the differentiation of abnormal location and direction caused by stem cell leakage often reduces the therapeutic effect, sometimes leading to serious consequences such as tumorigenesis. Therefore, the introduction of biomaterials cannot only prevent stem cell leakage but also provide suitable spatial structure for stem cells. In addition, the combination of drugs can provide further support for stem cells. Bertolo *et al.* (Bertolo et al., 2015), developed an injectable microcarrier with a size between 100 and 1,500 µm based on collagen, which achieved significant cell proliferation under 5% hypoxia. Andrea *et al.* (Friedmann et al., 2021), implanted collagen-based scaffolds loaded with ADMSCs into a sheep IDD model and made observations for up to 1 year. The researchers noted a stabilization of disc degeneration but did not observe recovery of disc height (Figure 2). Similarly, Zhang *et al.* (Zhang et al., 2014), used chitosan and alginate to fabricate an injectable 3D scaffold and achieved good growth and differentiation of ADMSCs under 2% hypoxia. Daisuke *et al.* (Ukeba et al., 2020), combined ultra-pure alginate-based gel with BMSCs. Compared with the gel-free control group, stem cells that were cultured *in vitro* showed higher expression of growth factors and ECM-related genes, and the stem cells combined with the gel group effectively induced IVD regeneration *in vivo*. Upon further comparison of four kinds of commercial scaffolds approved for medical use, Alessandro *et al.* (Bertolo et al., 2012), indicated that collagen and gelation-based scaffolds had better cell survival rate and aggrecan expression. Peroglio *et al.* (Peroglio et al., 2013), developed a hyaluronic acid-based thermoreversible hydrogel, which showed better differentiation induction than the alginate *in vitro* culture. The hydrogel maintained 90% of MSCs viability in nucleotomized IVD for at least 1 week. To compensate for the lack of cell binding sites in alginate-based materials and the unsatisfactory mechanical properties of collagen-based materials, Guillaume *et al.* (Guillaume et al., 2015), developed an alginate-collagen composite scaffold with the property of shape memory. In addition, the composite scaffold showed better cell fixation ability than alginate only scaffold and maintained the viability of BMSCs for 5 weeks.

**FIGURE 3:**
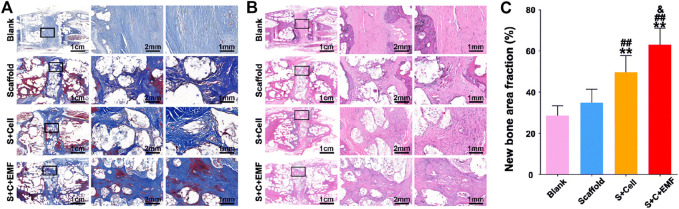
Masson trichrome staining **(A)** and HE staining **(B)** were used to observe the new bone formation in the scaffolds after 12 weeks of implantation. Quantification analysis of new bone fraction in four groups after 12 weeks of implantation (*n* = 6) **(C)**. ** means *p* < 0.01 compared to the blank group, ^##^ means *p* < 0.01 compared to the only scaffold group, ^&^means *p* < 0.05 compared to the S + Cell group. EMF: electromagnetic field. Reproduced with permission from (Li et al., 2021).

The high pressure in human IVD may cause the displacement of liquid cell carrier, which has higher requirements on the mechanical properties of the material. Zeng *et al.* (Zeng et al., 2015), loaded the alginate precursor encapsulated with ADMSCs into Poly (ethylene glycol) diacrylate (PEGDA). This thermos-responsive hydrogel improved cell survival rate while preventing cell leakage. Moreover, a better reduction in degeneration was observed than in other treatment groups in an IDD dog model after 6 months. In 2017, Diba *et al.* (Diba et al., 2017), assembled silica and gelatin nanoparticles to form a colloidal gel with excellent mechanical properties and impressive self-healing ability upon shear failure. In their subsequent study, they used this colloidal gel as the carrier to deliver BMSCs and injected it into rabbit IDD models. The gel showed excellent biocompatibility and degradability *in vivo*. Besides, the colloidal gel effectively prevented cell leakage, in addition to providing a favorable environment for the growth and differentiation of the BMSCs. Apart from the characteristics of the material itself, external physical stimulation can also affect stem cells. Elsaadany *et al.* (Elsaadany et al., 2017), evaluated the effects of different equiaxial mechanical strains and frequencies on the survival of ADMSCs in the scaffold and indicated that under suitable loading, ECM protein secretion and AD marker gene expression were significantly increased. Similarly, Li *et al.* (Li et al., 2021), used poly-caprolactone and nano-hydroxyapatite to fabricate scaffolds to load BMSCs, prior to treating these scaffolds with a sinusoidal electromagnetic field. The results indicated that stimulated BMSCs exhibited excellent osteogenic potential (Figure 3). Artificial polymer materials are easy to work with so they can be used to determine the relationship between material properties and stem cells. Tasi et al. (Tsai et al., 2014), used poly-l-lactic acid and poly-caprolactone to construct a fibrous scaffold mimicking the AF structure. Zhu et al. (Zhu et al., 2016a), used a biodegradable poly (ether carbonate urethane) urea material to achieve adjustable scaffold elasticity. AFSCs exhibited significant differences in protein expression on different elastic scaffolds. Chu et al. (Chu et al., 2019), further used this scaffold to demonstrate that the mechanical properties, topography, and geometric characteristics of the material affect the growth and differentiation of AFSCs.

**FIGURE 4:**
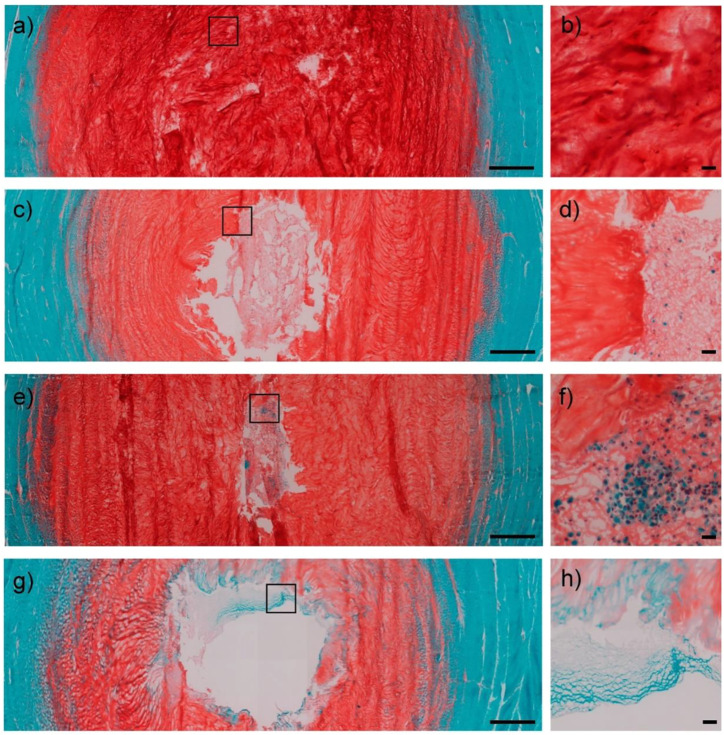
IVD cross-sections were visualized using safranin O/fast green staining. Healthy disc controls in low (1.25×) **(A)** and high (20×) **(B)** magnification images. Low cell density hydrogel (1 million MSCs) in low (1.25×) **(C)** and high (20×) **(D)** magnification images. High cell density hydrogel (2 million MSCs) in low (1.25×) **(E)** and high (20×) **(F)** magnification images. Only hydrogel group in low (1.25×) **(G)** and high (20×) **(H)** magnification images. Scale bar = 1,000 μm (low magnification); 50 μm (high magnification). Reproduced with permission from (Russo et al., 2021).

Adding cytokines or drugs to the material offers more possibilities to control stem cell growth and differentiation in the material. TGF is an important signal factor for IVD development and repair, considering that it can stimulate ECM anabolism, induce stem cell differentiation, and inhibit inflammatory response and ECM catabolism (Watabe and Miyazono, 2009; Yang et al., 2015; Chen et al., 2019). Liang *et al.* (Liang et al., 2013), reported a cell carrier by adding nanoparticles containing dexamethasone and TGF-β3 to poly (lactide-co-glycolide) microspheres loaded with ADMSCs. At week 24 post-transplantation, the mice models showed significant disc height recovery and proteoglycan accumulation. Similar results were reported by Kim *et al.*, (Kim M. J. et al., 2020), who engineered porous particles with leaf-stack structures to simultaneously load BMSCs and TGF-β3; such particles released TGF-β3 continuously for up to 18 days. As a result, significant disc regeneration was observed in dog IDD models. To further overcome the defects of rapid clearance and short half-life of TGF *in vivo*, Shen *et al.* (Shen et al., 2020), used graphene oxide nanosheets to achieve slow release of TGF. Whether cultured *in vitro* or implanted subcutaneously, the addition of this material caused BMSCs in the hydrogel to express more cartilage matrix. In addition, the initial compression strength of the hydrogel was also enhanced by graphene oxide nanosheets. Furthermore, the repair stimulatory effect of TGF on the NP and AF cells has also been demonstrated (Guillaume et al., 2014; Ligorio et al., 2021). Platelet-rich plasma (PRP) obtained by autologous whole blood centrifugation can provide a greater variety of bioactive factors than a single TGF component, and its application in regeneration medicine has received extensive attention in recent years (Everts et al., 2020; Everts et al., 2021). Chen *et al.* (Chen et al., 2009), used the culture system containing PRP to culture MSCs and observed the upregulation of genes that are related to type II collagen, aggrecan, and an increased cartilage matrix deposition *in vivo*. Wang *et al.* (Wang et al., 2018), further compared the effects of different doses of PRP and the presence or absence of leukocytes in PRP on NP stem cells, and at a 10% dose, PRP without leukocytes exerted the best differentiation effects on NP stem cells. Russo *et al.* (Russo et al., 2021), further developed a hydrogel composed of PRP and hyaluronic acid as the carrier to deliver the BMSCs, and this could integrate well with the surrounding tissue while maintaining cell viability (Figure 4).

**FIGURE 5:**
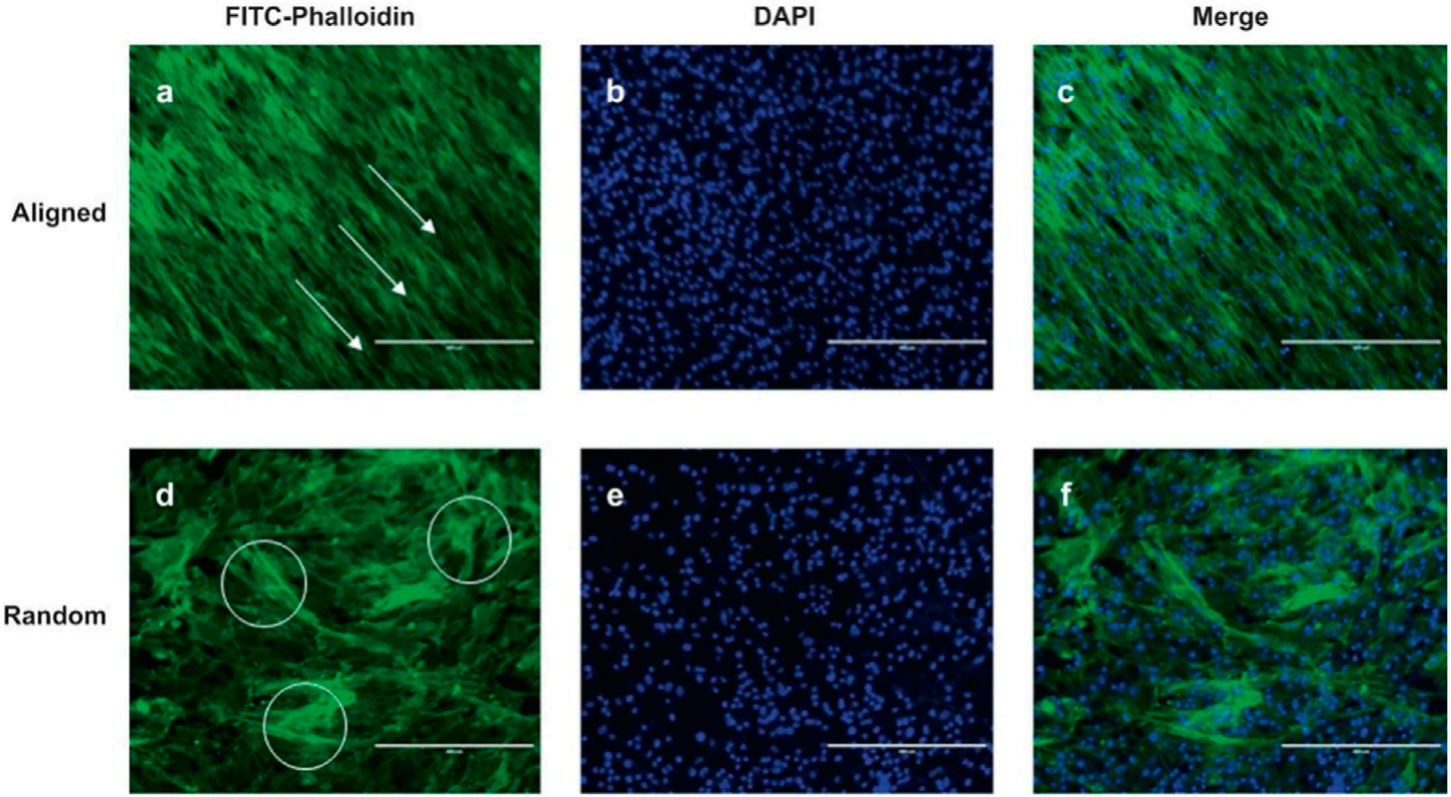
AF stem cell cytoskeletal images of aligned **(A–C)** and random **(D–F)** scaffolds were processed using FITC-phalloidin (green) and DAPI (blue) staining. White arrow and circle are the selected represent area. Scale bar = 400 μm. DAPI (4′,6-diamidino-2-phenylindole). Reproduced with permission from (Liu et al., 2015a).

New tissue engineering technologies allow people to design and manipulate material properties more accurately. In recent years, an emerging technology called 3D bioprinting has attracted much attention in tissue engineering, a scenario that improves the control of the spatial distribution of materials to a new level (Shapira and Dvir, 2021; Zhu et al., 2016b; D'Este et al., 2018). Serra *et al.* (Serra et al., 2016), used 3D bioprinting to design an anatomical lumbar spine skeleton with similar mechanical properties to trabecular bone and demonstrated ideal biocompatibility in preliminary cell culture. Tarafder *et al.* (Tarafder et al., 2016), achieved precise spatial control of growth factor release by exploiting the differences in the melting points of various materials, in addition to interchanging dispensing cartridges during a single printing process. Electrospinning is a technology that can be used to produce ultrathin fibers. It makes it possible to reduce the diameter of ultrathin fibers to nanometer levels (Hong et al., 2019; Xue et al., 2019). Liu *et al.* (Liu et al., 2015a), fabricated an aligned fiber-polyurethane scaffold carrying AF stem cells using the electrospinning technology, and the AFSCs on this kind of scaffold were more aligned and produced more collagen Ⅰ and aggrecan (Figure 5). Wang *et al.* (Wang et al., 2020), compared the effects of static culture, intermittent centrifugation culture, and dynamic bioreactor on the infiltration ability of BMSCs in low-porosity electrospinning scaffolds. Zhu *et al.* (Zhu et al., 2021), combined 3D printing and electrospinning technology to construct a composite scaffold, which simulate the structure and properties of the NP and AF at the same time. The BMSCs that are distributed in the scaffold maintain ideal cell viability. In an *in vivo* experiment, new ECM production was observed and IVD height was maintained.

### 3.3 New directions based on stem cells

**FIGURE 6:**
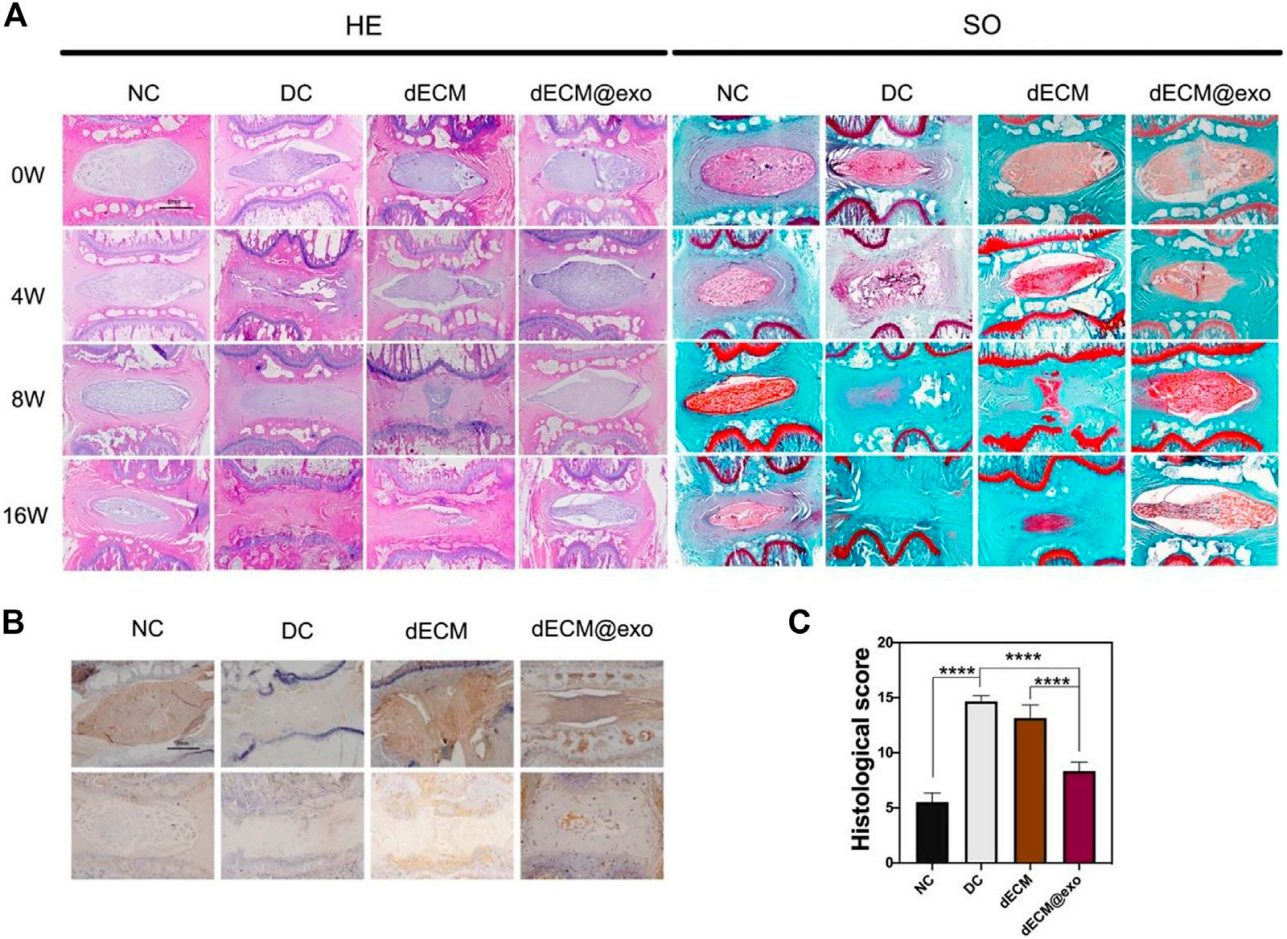
Images of each group were obtained by H&E and saffron O staining method at different times **(A)**. Scale bar = 1 mm. Immunohistochemical images of aggrecan and collagen II were examined **(B)**. Quantitative analysis of histological grade in each group at 16 weeks **(C)**. *means *p* < 0.05. NC: normal disc group. DC: degenerated disc group. dECM: only the hydrogel was injected. dECM@exo: hydrogel containing exosomes were injected. Reproduced with permission from (Xing et al., 2021).

Considering the unavoidable ethical and cell survival problems of stem cell therapy, extracellular vesicles based on stem cells have attracted attention as a new treatment method for IDD in recent years. Extracellular vesicles can be roughly divided into exosomes, microvesicles, and apoptotic bodies, according to their size. Exosomes are the smallest category, with a size of about 30–150 nm (Vader et al., 2016; Li et al., 2019). Apoptotic bodies are the largest, with a size of between 50 nm and 5 μm. Microvescicles are in the middle with a size of about 50–100 nm. Of all the extracellular vesicles, exosomes have been the most extensively studied. Almost all cells will produce exosomes, the outer layer is the lipid double layer, which contains a variety of substances like cytokines, proteins, lipids, mRNA, among others (DiStefano et al., 2022a; Bhujel et al., 2022). Due to their cell origin, exosomes have extremely low immunogenicity. As a carrier of intercellular information, exosomes can act on a variety of signal transduction pathways, such as fusion, endocytosis, and soluble signaling pathways (Zhang et al., 2019). A remarkable number of studies confirmed that stem cell-derived exosomes interfere with the pathological development of IDD through various mechanisms. Liao et al. (Liao et al., 2019), demonstrated that exosome-derived BMSCs could ameliorate excessive apoptosis in IDD, through endoplasmic reticulum stress. Similarly, Xiang *et al.* (Xiang et al., 2020), indicated that urine-derived stem cells could also regulate endoplasmic reticulum stress. Zhu et al. (Guo et al., 2021), further demonstrated that exosomes from urine-derived stem cells have the effect of promoting cell proliferation and ECM synthesis by regulating TGF-β. In an *in vivo* experiment using a rabbit IDD model, exosomes derived from MSCs also exhibited anti-oxidative and anti-inflammatory effects and prevented further degeneration. The combination of exosomes and biomaterials also has a broad application prospect. Luo *et al.* (Luo et al., 2022), designed a cartilage ECM hydrogel that was loaded with CEP stem cells and injected the hydrogel near the CEPs in mouse models. They observed that the exosomes produced by the stem cells penetrated the AFs to reach the NP cells and attenuated the development of IDD. Xing *et al.* (Xing et al., 2021), designed a thermosensitive hydrogel loaded with ADSC exosomes. The hydrogel provides a suitable environment for the growth of NP cells, and the exosomes regulate the anabolism and catabolism of the ECM by regulating metalloproteinases (Figure 6). DiStefano *et al.* (DiStefano et al., 2022b), loaded exosomes produced by MSCs under different oxygen concentrations into poly (lactic-co-glycolic acid) microspheres and showed that AF cells were more sensitive to the production of exosomes under hypoxic conditions.

Generally, the exosome strategy avoids many problems that are difficult for stem cells to circumvent while retaining most of the advantages of the stem cell strategy. Although exosomes brought a new direction to regenerative medicine, their application in IDD is still in its infancy. Its formal application in clinical practice still requires extensive evaluation. The metabolism, distribution, appropriate dosage, and administration frequency of exosomes in the human body still need a lot of research. In addition, the differences in the expression of genetic information between different individuals may lead to significant variations in the therapeutic effects of exosomes. Therefore, a comprehensive and multi-level evaluation system is necessary. From the point of view of drug production, their large-scale preparation is still difficult, not to talk of a lack of unified standards. The storage and transportation of exosome also need to be considered.

## 4 Conclusion

The improvement effect of stem cells combined with biomaterials on IDD has been fully proved through a wealth of experiments. However, there are still many problems that are worth considering besides the benefits that comes with the application of the “stem cell-biomaterial” combo the human body. First, the structure of the IVD and the types of functional cells that are involved are complex. The pathological mechanism of IDD is also multifaceted. It is often difficult to obtain satisfactory results for the repair and supplementation of a tissue or cell. Second, despite the increasing understanding of various stem cells and the continuous developments in tissue engineering, choosing a personalized match according to the actual situation of patients still requires extensive pre-clinical testing. In addition to long-standing issues such as safety, cell origin, and ethics, large-scale preparation and production costs are key factors in that need to be carefully considered in practical applications. Finally, considering that IDD is not always accompanied by obvious symptoms, the procedure for screening patients with early IDD in a manner that is comprehensive enough to persuade them to employ timely interventions is a problem that needs to dealt with in clinical application.

Future research may focus on the following aspects: 1) A deeper understanding of the pathological mechanism of IDD and more effective therapeutic targets will provide more succinct directions for selecting drugs and preparing materials; 2) Further exploration of stem cell repair mechanisms, as well as efficient and stable differentiation induction methods; 3) Identification of materials with good properties that can withstand different mechanical requirements in various areas of IVD, in addition to providing a suitable microenvironment for cells; 4) Development of IDD models that closely imitate human IVD environment development and the corresponding *in vivo* experiments; 5) Earlier, faster, more economical screening techniques for IDD, as well as comprehensive and easy-to-accept patient education.
